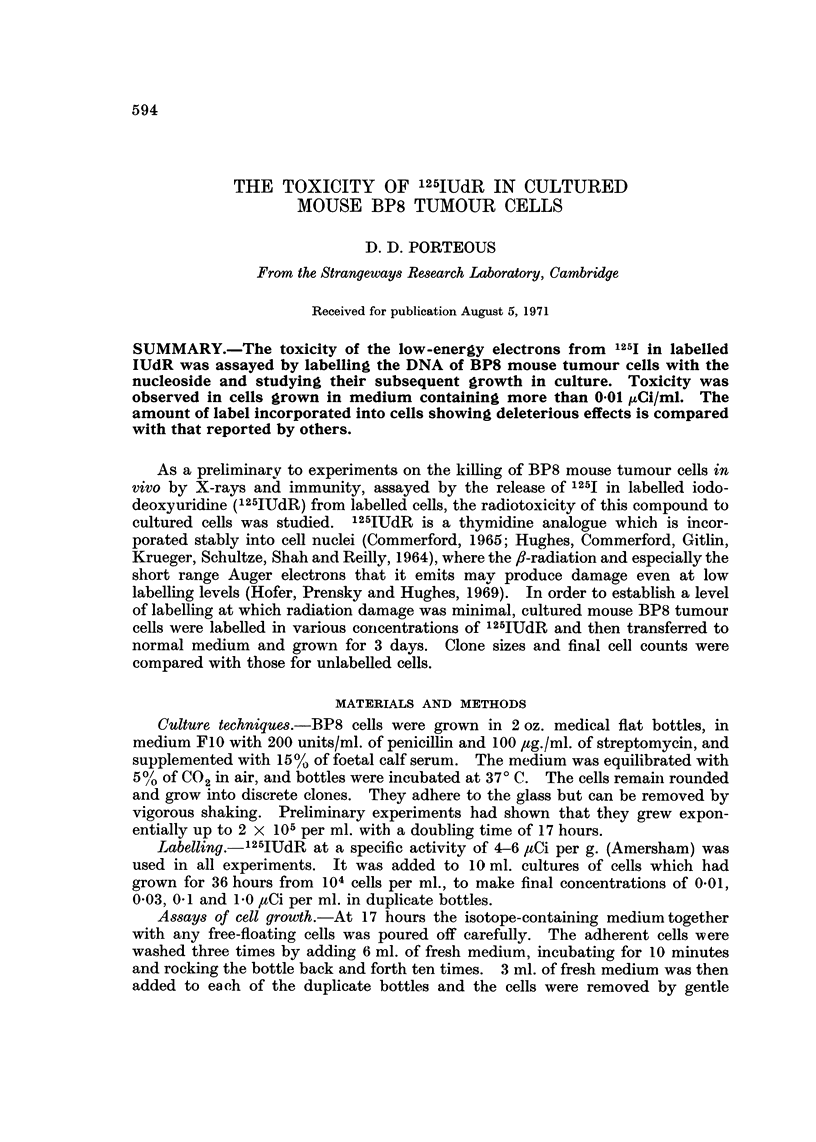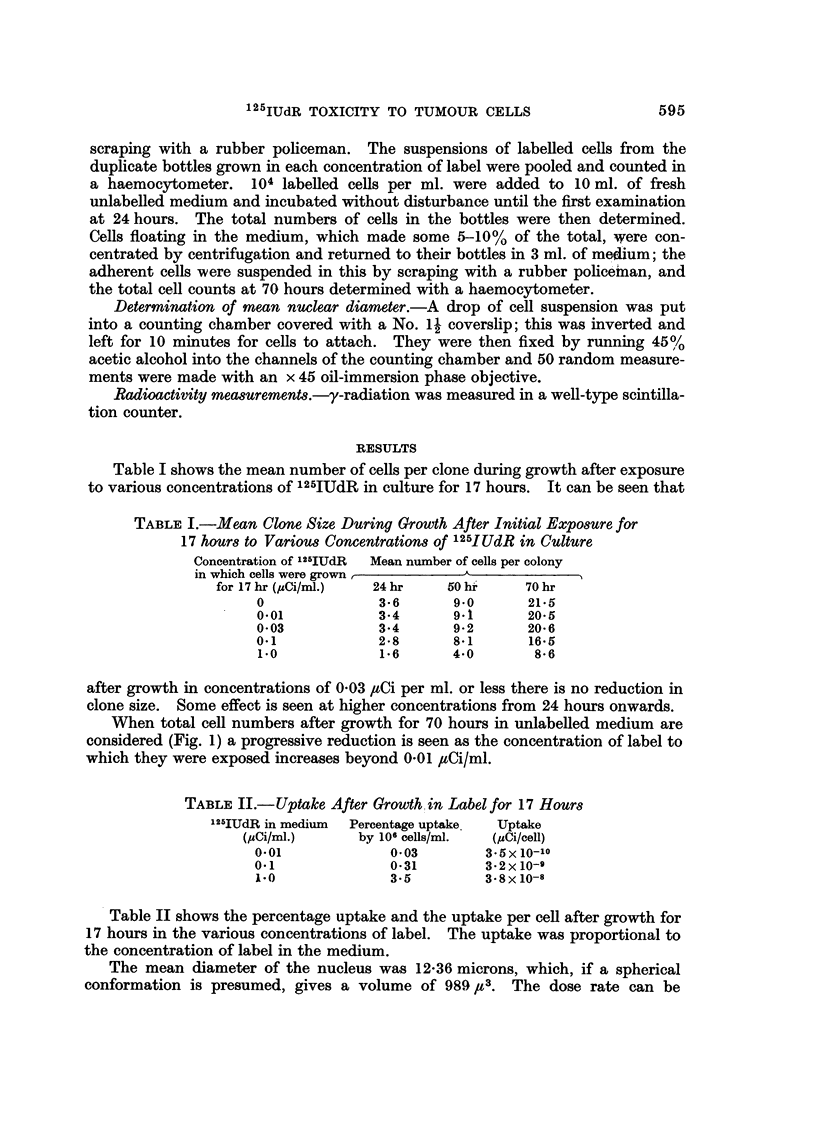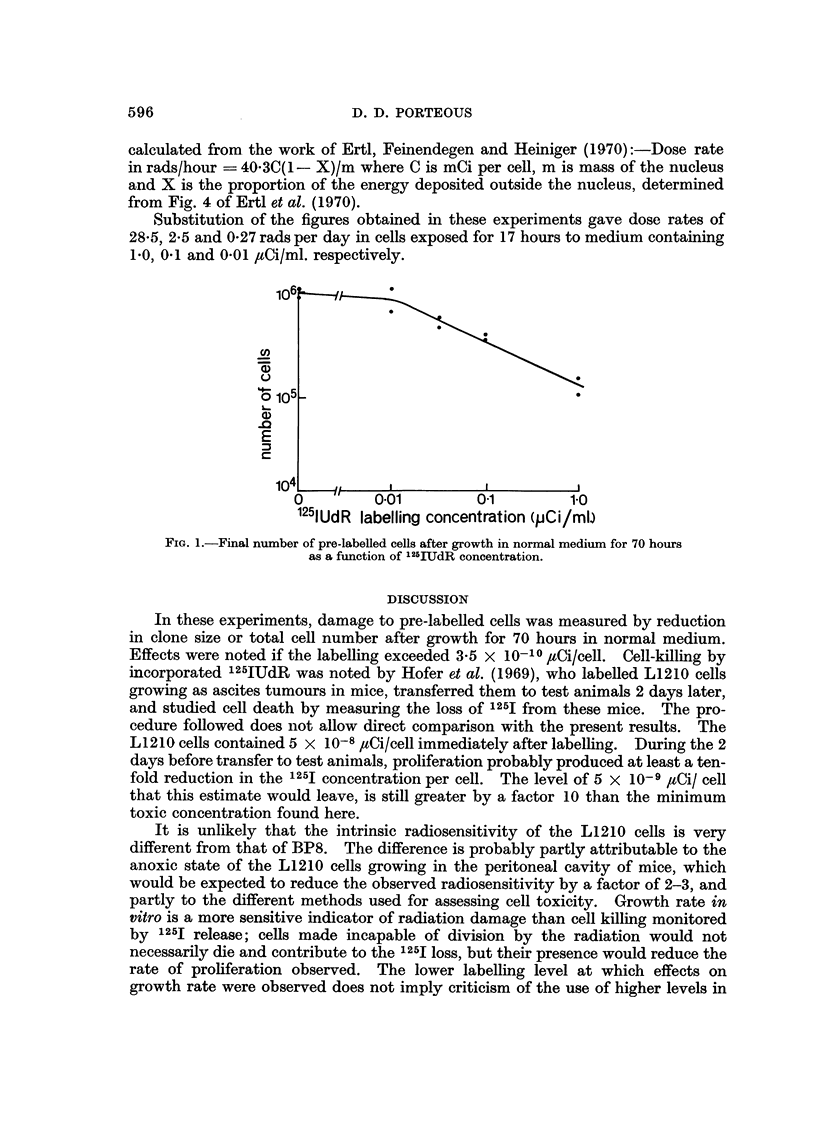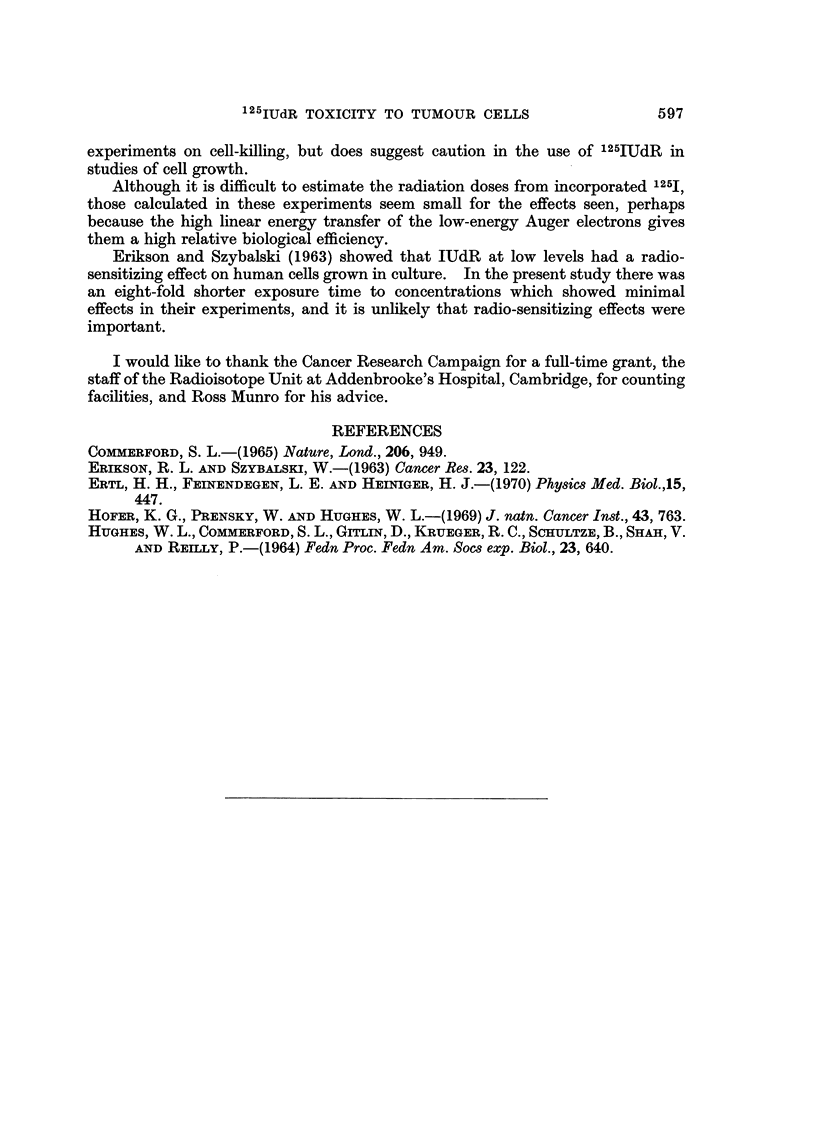# The Toxicity of ^125^IUdR in Cultured Mouse BP8 Tumour Cells

**DOI:** 10.1038/bjc.1971.75

**Published:** 1971-09

**Authors:** D. D. Porteous

## Abstract

The toxicity of the low-energy electrons from ^125^I in labelled IUdR was assayed by labelling the DNA of BP8 mouse tumour cells with the nucleoside and studying their subsequent growth in culture. Toxicity was observed in cells grown in medium containing more than 0·01 μCi/ml. The amount of label incorporated into cells showing deleterious effects is compared with that reported by others.


### THE TOXICITY OF $^{125}$IUdR IN CULTURED MOUSE BP8 TUMOUR CELLS

D. D. PORTEOUS

*From the Strangeways Research Laboratory, Cambridge*

Received for publication August 5, 1971

**SUMMARY.—The toxicity of the low-energy electrons from $^{125}$I in labelled IUdR was assayed by labelling the DNA of BP8 mouse tumour cells with the nucleoside and studying their subsequent growth in culture. Toxicity was observed in cells grown in medium containing more than 0·01 μCi/ml. The amount of label incorporated into cells showing deleterious effects is compared with that reported by others.**

As a preliminary to experiments on the killing of BP8 mouse tumour cells *in vivo* by X-rays and immunity, assayed by the release of $^{125}$I in labelled iododeoxyuridine ($^{125}$IUdR) from labelled cells, the radiotoxicity of this compound to cultured cells was studied. $^{125}$IUdR is a thymidine analogue which is incorporated stably into cell nuclei (Commerford, 1965; Hughes, Commerford, Gitlin, Krueger, Schultze, Shah and Reilly, 1964), where the β-radiation and especially the short range Auger electrons that it emits may produce damage even at low labelling levels (Hofer, Prensky and Hughes, 1969). In order to establish a level of labelling at which radiation damage was minimal, cultured mouse BP8 tumour cells were labelled in various concentrations of $^{125}$IUdR and then transferred to normal medium and grown for 3 days. Clone sizes and final cell counts were compared with those for unlabelled cells.

### MATERIALS AND METHODS

*Culture techniques.*—BP8 cells were grown in 2 oz. medical flat bottles, in medium F10 with 200 units/ml. of penicillin and 100 μg./ml. of streptomycin, and supplemented with 15% of foetal calf serum. The medium was equilibrated with 5% of $CO_2$ in air, and bottles were incubated at 37° C. The cells remain rounded and grow into discrete clones. They adhere to the glass but can be removed by vigorous shaking. Preliminary experiments had shown that they grew exponentially up to $2 \times 10^5$ per ml. with a doubling time of 17 hours.

*Labelling.*—$^{125}$IUdR at a specific activity of 4–6 μCi per g. (Amersham) was used in all experiments. It was added to 10 ml. cultures of cells which had grown for 36 hours from $10^4$ cells per ml., to make final concentrations of 0·01, 0·03, 0·1 and 1·0 μCi per ml. in duplicate bottles.

*Assays of cell growth.*—At 17 hours the isotope-containing medium together with any free-floating cells was poured off carefully. The adherent cells were washed three times by adding 6 ml. of fresh medium, incubating for 10 minutes and rocking the bottle back and forth ten times. 3 ml. of fresh medium was then added to each of the duplicate bottles and the cells were removed by gentle scraping with a rubber policeman. The suspensions of labelled cells from the duplicate bottles grown in each concentration of label were pooled and counted in a haemocytometer. $10^4$ labelled cells per ml. were added to 10 ml. of fresh unlabelled medium and incubated without disturbance until the first examination at 24 hours. The total numbers of cells in the bottles were then determined. Cells floating in the medium, which made some 5–10% of the total, were concentrated by centrifugation and returned to their bottles in 3 ml. of medium; the adherent cells were suspended in this by scraping with a rubber policeman, and the total cell counts at 70 hours determined with a haemocytometer.

*Determination of mean nuclear diameter.*—A drop of cell suspension was put into a counting chamber covered with a No. 1½ coverslip; this was inverted and left for 10 minutes for cells to attach. They were then fixed by running 45% acetic alcohol into the channels of the counting chamber and 50 random measurements were made with an ×45 oil-immersion phase objective.

*Radioactivity measurements.*—$\gamma$-radiation was measured in a well-type scintillation counter.

### RESULTS

Table I shows the mean number of cells per clone during growth after exposure to various concentrations of $^{125}$IUdR in culture for 17 hours. It can be seen that

TABLE I.—*Mean Clone Size During Growth After Initial Exposure for 17 hours to Various Concentrations of $^{125}$IUdR in Culture*

| Concentration of $^{125}$IUdR in which cells were grown for 17 hr ($\mu$Ci/ml.) | Mean number of cells per colony | | |
|---|---|---|---|
| | 24 hr | 50 hr | 70 hr |
| 0 | 3·6 | 9·0 | 21·5 |
| 0·01 | 3·4 | 9·1 | 20·5 |
| 0·03 | 3·4 | 9·2 | 20·6 |
| 0·1 | 2·8 | 8·1 | 16·5 |
| 1·0 | 1·6 | 4·0 | 8·6 |

after growth in concentrations of 0·03 $\mu$Ci per ml. or less there is no reduction in clone size. Some effect is seen at higher concentrations from 24 hours onwards.

When total cell numbers after growth for 70 hours in unlabelled medium are considered (Fig. 1) a progressive reduction is seen as the concentration of label to which they were exposed increases beyond 0·01 $\mu$Ci/ml.

TABLE II.—*Uptake After Growth in Label for 17 Hours*

| $^{125}$IUdR in medium ($\mu$Ci/ml.) | Percentage uptake by $10^6$ cells/ml. | Uptake ($\mu$Ci/cell) |
|---|---|---|
| 0·01 | 0·03 | $3·5 \times 10^{-10}$ |
| 0·1 | 0·31 | $3·2 \times 10^{-9}$ |
| 1·0 | 3·5 | $3·8 \times 10^{-8}$ |

Table II shows the percentage uptake and the uptake per cell after growth for 17 hours in the various concentrations of label. The uptake was proportional to the concentration of label in the medium.

The mean diameter of the nucleus was 12·36 microns, which, if a spherical conformation is presumed, gives a volume of 989 $\mu^3$. The dose rate can be calculated from the work of Ertl, Feinendegen and Heiniger (1970):—Dose rate in rads/hour $= 40\cdot3C(1 - X)/m$ where C is mCi per cell, m is mass of the nucleus and X is the proportion of the energy deposited outside the nucleus, determined from Fig. 4 of Ertl *et al.* (1970).

Substitution of the figures obtained in these experiments gave dose rates of 28·5, 2·5 and 0·27 rads per day in cells exposed for 17 hours to medium containing 1·0, 0·1 and 0·01 $\mu$Ci/ml. respectively.

FIG. 1.—Final number of pre-labelled cells after growth in normal medium for 70 hours as a function of $^{125}$IUdR concentration.

DISCUSSION

In these experiments, damage to pre-labelled cells was measured by reduction in clone size or total cell number after growth for 70 hours in normal medium. Effects were noted if the labelling exceeded $3\cdot5 \times 10^{-10}$ $\mu$Ci/cell. Cell-killing by incorporated $^{125}$IUdR was noted by Hofer *et al.* (1969), who labelled L1210 cells growing as ascites tumours in mice, transferred them to test animals 2 days later, and studied cell death by measuring the loss of $^{125}$I from these mice. The procedure followed does not allow direct comparison with the present results. The L1210 cells contained $5 \times 10^{-8}$ $\mu$Ci/cell immediately after labelling. During the 2 days before transfer to test animals, proliferation probably produced at least a tenfold reduction in the $^{125}$I concentration per cell. The level of $5 \times 10^{-9}$ $\mu$Ci/ cell that this estimate would leave, is still greater by a factor 10 than the minimum toxic concentration found here.

It is unlikely that the intrinsic radiosensitivity of the L1210 cells is very different from that of BP8. The difference is probably partly attributable to the anoxic state of the L1210 cells growing in the peritoneal cavity of mice, which would be expected to reduce the observed radiosensitivity by a factor of 2–3, and partly to the different methods used for assessing cell toxicity. Growth rate *in vitro* is a more sensitive indicator of radiation damage than cell killing monitored by $^{125}$I release; cells made incapable of division by the radiation would not necessarily die and contribute to the $^{125}$I loss, but their presence would reduce the rate of proliferation observed. The lower labelling level at which effects on growth rate were observed does not imply criticism of the use of higher levels in experiments on cell-killing, but does suggest caution in the use of $^{125}$IUdR in studies of cell growth.

Although it is difficult to estimate the radiation doses from incorporated $^{125}$I, those calculated in these experiments seem small for the effects seen, perhaps because the high linear energy transfer of the low-energy Auger electrons gives them a high relative biological efficiency.

Erikson and Szybalski (1963) showed that IUdR at low levels had a radio-sensitizing effect on human cells grown in culture. In the present study there was an eight-fold shorter exposure time to concentrations which showed minimal effects in their experiments, and it is unlikely that radio-sensitizing effects were important.

I would like to thank the Cancer Research Campaign for a full-time grant, the staff of the Radioisotope Unit at Addenbrooke's Hospital, Cambridge, for counting facilities, and Ross Munro for his advice.

### REFERENCES

Commerford, S. L.—(1965) *Nature, Lond.*, **206**, 949.
Erikson, R. L. and Szybalski, W.—(1963) *Cancer Res.* **23**, 122.
Ertl, H. H., Feinendegen, L. E. and Heiniger, H. J.—(1970) *Physics Med. Biol.*,**15**, 447.
Hofer, K. G., Prensky, W. and Hughes, W. L.—(1969) *J. natn. Cancer Inst.*, **43**, 763.
Hughes, W. L., Commerford, S. L., Gitlin, D., Krueger, R. C., Schultze, B., Shah, V. and Reilly, P.—(1964) *Fedn Proc. Fedn Am. Socs exp. Biol.*, **23**, 640.